# MiR-125b regulates proliferation and apoptosis of nasopharyngeal carcinoma by targeting A20/NF-κB signaling pathway

**DOI:** 10.1038/cddis.2017.211

**Published:** 2017-06-01

**Authors:** Zhen Zheng, Jia-Quan Qu, Hong-Mei Yi, Xu Ye, Wei Huang, Ta Xiao, Jiao-Yang Li, Yuan-Yuan Wang, Juan Feng, Jin-Feng Zhu, Shan-Shan Lu, Hong Yi, Zhi-Qiang Xiao

**Affiliations:** 1 Research Center of Carcinogenesis and Targeted Therapy, Xiangya Hospital, Central South University, Changsha, Hunan 410008, China; 2 The Higher Educational Key Laboratory for Cancer Proteomics and Translational Medicine of Hunan Province, Xiangya Hospital, Central South University, Changsha, Hunan 410008, China; 3 Department of Oncology, Qianjiang Central Hospital of Chongqing, Jishou University, Hunan 416000, China; 4 Department of Dermatology, Xiangya Hospital, Central South University, Changsha, Hunan 410008, China

## Abstract

MiR-125b is aberrantly expressed and has a role in the various types of tumors. However, the role and mechanism of miR-125b in nasopharyngeal carcinoma (NPC) are unclear. In this study, we investigated the role and mechanism of miR-125b in NPC. We observed that miR-125b was significantly upregulated in the NPC tissues relative to normal nasopharyngeal mucosa (NNM), and its increment was correlated with poor patient survival, and was an independent predictor for reduced patient survival; miR-125b promoted NPC cell proliferation and inhibited NPC cell apoptosis; in a mouse model, administration of miR-125b antagomir significantly reduced the growth of NPC xenograft tumors. Mechanistically, we confirmed that A20 was a direct target of miR-125b, and found that activation of nuclear factor *κ*B (NF-*κ*B) signaling pathway by A20 mediated miR-125b-promoting NPC cell proliferation and -inhibiting NPC cell apoptosis. With a combination of loss-of-function and gain-of-function approaches, we further showed that A20 inhibited NPC cell proliferation, induced NPC cell apoptosis, and reduced the growth of NPC xenograft tumors. Moreover, A20 was significantly downregulated, whereas p-p65(RelA) was significantly upregulated in the NPC tissues relative to normal nasopharyngeal mucosa, and miR-125b level was negatively associated with A20 level, whereas positively associated with p-p65 level. Our data demonstrate that miR-125b regulates NPC cell proliferation and apoptosis by targeting A20/NF-κB signaling pathway, and miR-125b acts as oncogene, whereas A20 functions as tumor suppressor in NPC, highlighting the therapeutic potential of miR-125b/A20/NF-κB signaling axis in the NPC.

MicroRNAs (miRNAs) are a class of non-coding RNAs that post-transcriptionally silence target mRNAs.^1^ MiRNAs have an important role in normal cell homeostasis, and dysregulation of miRNAs expression has been implicated in human cancers.^2, 3^ One miRNA that has gained special interest in the field of cancer research is miR-125b.^4^ In some tumor types, miR-125b is upregulated and displays oncogenic potential, as it induces cell proliferation and inhibits cell apoptosis, contributing to malignant transformation.^5, 6, 7^ In other tumors, miR-125b is downregulated and accompanied by de-repression of cellular proliferation and anti-apoptotic programs, corresponding to the loss of tumor suppressive functions.^5, 6, 7^ Although miR-125b is aberrantly expressed in a great variety of tumors and acts as oncogene or tumor suppressor, the expression and function of miR-125b in nasopharyngeal carcinoma (NPC) are unclear.

Nuclear factor *κ*B (NF-*κ*B) is an important regulator of proliferation and apoptosis.^8^ Activation of NF-*κ*B signaling pathway has a crucial role in the development and progression of NPC.^9, 10, 11, 12^ NF-κB comprises two subunits, commonly p50(NFκB1)/p65(RelA), which in its inactive state is held in the cytoplasm by the inhibitor of NF-*κ*B (I*κ*B). I*κ*B is regulated by I*κ*B kinases (IKKs): upon stimulation by external signals or stress, IKK is activated and phosphorylates I*κ*B, and thereby targets I*κ*B to ubiquitin-mediated protein degradation. As a result, NF-*κ*B is released and translocates into the nucleus where it transactivates multiple genes involved in proliferation and apoptosis.^8, 13^

Ubiquitin modification, occurring at multiple steps within the NF-*κ*B signaling cascades, serves as a regulator in NF-*κ*B activation.^14^ A growing number of proteins, such as receptor-interacting protein kinase (RIP-1), TRAF2, TRAF6, and NEMO, in the NF-*κ*B signal pathway have been identified to be modified by ubiquitin.^14, 15, 16, 17^ Tumor necrosis factor alpha-induced protein 3 (TNFAIP3, also known as A20), functioning as an ubiquitin-editing enzyme,^15^ negatively regulates NF-*κ*B signaling through dual mechanisms*,* that is, deconjugation of K63-linked polyubiquitin chains from RIP-1 and subsequent conjugation of RIP-1 with K48-linked polyubiquitin chains for proteasomal degradation.^16, 17^ A20 can also catalyze the cleavage of K63-linked ubiquitin chains and the conjugation of K48-linked polyubiquitin chains, thereby targeting TRAF2, TRAF6 and NEMO for proteasomal degradation.^18, 19^ Therefore, A20 serves as a negative regulator in NF-*κ*B signal pathway by inhibiting the activity of its upstream signaling transducers.

Several studies have reported that miR-125b activates NF-*κ*B pathway by targeting A20.^20, 21, 22^ Therefore, we hypothesize that miR-125b is dysregulated in NPC, and has a crucial role in the pathogenesis of NPC by targeting A20 and then regulating NF-κB signaling activity. Here, we report that miR-125b is upregulated, whereas A20 is downregulated in NPC tissues relative to normal nasopharyngeal mucosa (NNM); miR-125b promotes NPC cell proliferation and inhibits NPC cell apoptosis by targeting A20/NF-κB signaling pathway; A20 inhibits NPC cell proliferation, induces NPC cell apoptosis, and reduces the growth of NPC xenograft tumors. Our data demonstrate that miR-125b acts as oncogene, whereas A20 functions as tumor suppressor in NPC, and suggest that NPC patients with high miR-125b expression might benefit from specific targeted therapies directed at miR-125b/A20/NF-κB signaling pathway.

## Results

### MiR-125b expression is increased in the NPC tissues and correlated with NPC patient prognosis

**Figure 1:**
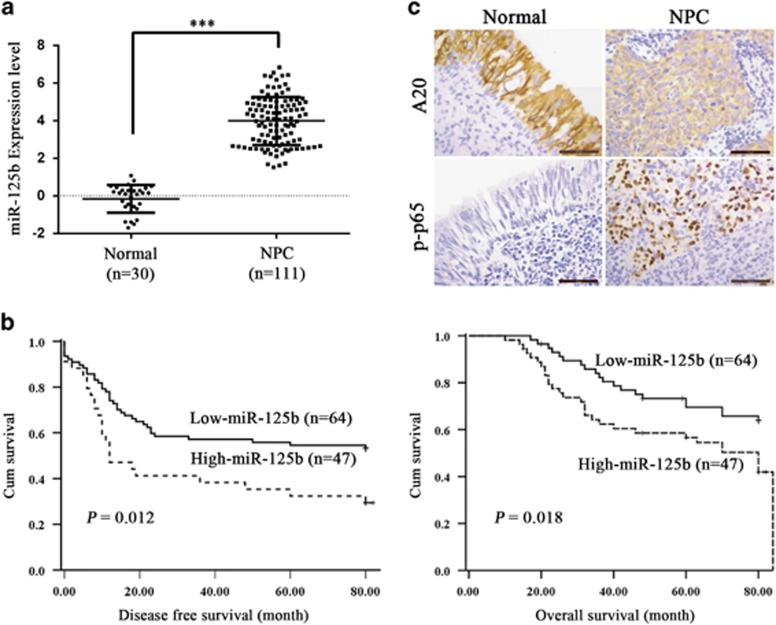
The expression of miR-125b, A20, and p-p65 in NPC and association of miR-125b expression levels with the patient survival. (**a**) QRT-PCR analysis of the expression levels of miR-125b in the 111 NPC tissues and 30 normal nasopharyngeal mucosa (NNM). Three experiments were done; Means, S.D.s, and statistical significance are denoted; ***P<0.001. (**b**) Kaplan–Meier survival analysis for 111 NPC patients according to the expression levels of miR-125b. NPC patients with high miR-125b expression have a significantly worse disease-free survival (left) and overall survival (right) than those with low miR-125b expression. The log-rank test was used to calculate *P*-value. (**c**) A representative result of immunohistochemistry showing the expression of A20 and p-p65 (RelA) in the NNM and NPC tissues. Scale bars=50 *μ*m

As the expression and significance of miR-125b in NPC are unclear, we detected miR-125b levels in the NPC tissues and NNM by using qRT-PCR. We observed that miR-125b expression was significantly increased in the NPC tissues as compared with NNM (Figure 1a). Survival analyses revealed that high miR-125b level in NPC tissues correlated with the markedly reduced disease-free survival (DFS) and overall survival (OS) of the patients (Figure 1b). A univariate and multivariate Cox regression analysis showed that high miR-125b expression was an independent predictor for the reduced DFS and OS of NPC patients (Supplementary Table S1). These results indicate the importance of high miR-125b expression in the NPC.

### MiR-125b promotes NPC cell proliferation and inhibits NPC cell apoptosis

**Figure 2:**
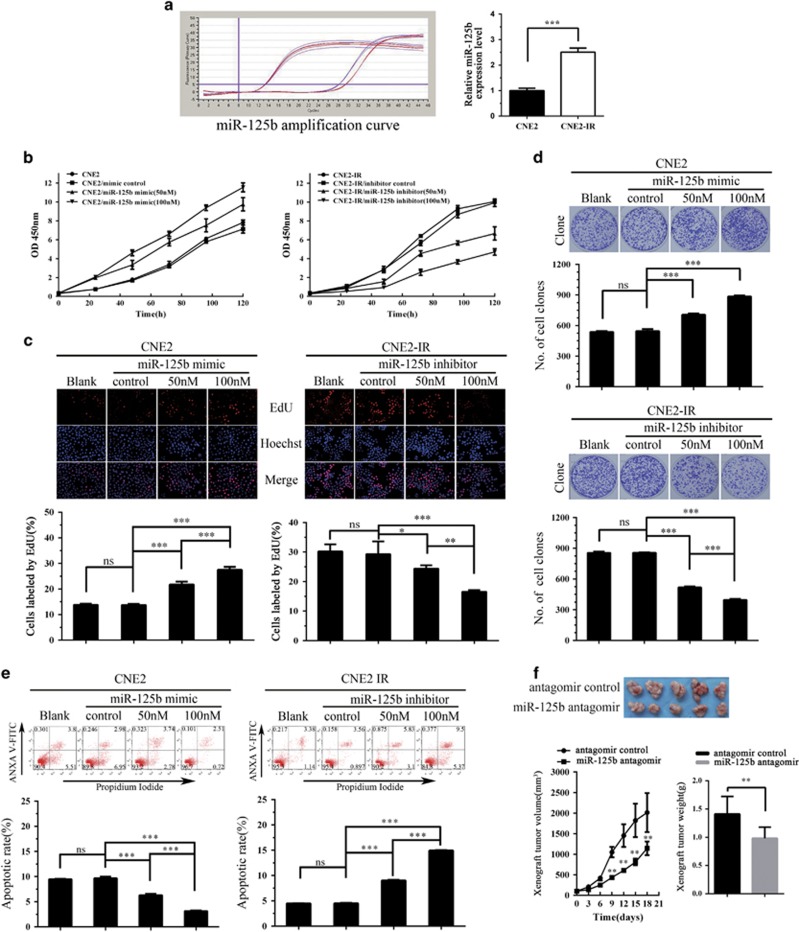
The effects of miR-125b on NPC cell proliferation, apoptosis, and xenograft growth. (**a**) QRT-PCR analysis of miR-125b expression levels in the NPC cell lines CNE2 and CNE2-IR. Analysis of cell proliferation by CCK-8 (**b**), EdU incorporation (**c**) and plate clone formation (**d**) assay in the miR-125b mimic-transfected CNE2 cells, miR-125b inhibitor-transfected CNE2-IR cells and their control cells. (**e**) Analysis of cell apoptosis by flow cytometry in the miR-125b mimic-transfected CNE2 cells, miR-125b inhibitor-transfected CNE2-IR cells and their control cells. (**f**) MiR-125b antagomir inhibits *in vivo* NPC cell growth. The photography of xenograft tumors after 18 days subcutaneous implantation of control or miR-125b antagomir-injected CNE2-IR CNE2 cells (top); growth and weight of the xenograft tumors (bottom). *n*=5 mice per group. Means, S.D.s, and statistical significance are denoted; *P<0.05; **P<0.01; ***P<0.001; ns, no significance

We used paired NPC cell lines, CNE2-IR and CNE2, to determine the effect of miR-125b on NPC cell proliferation and apoptosis. CNE2-IR cell line was derived from NPC CNE2 cell line, both cell lines have the same genetic background but different radiosensitivity, and CNE2-IR is radioresistant, whereas CNE2 is radiosenstive.^23^ Because miR-125b expression was significantly higher in the CNE2-IR cells than that in the CNE2 cells (Figure 2a), miR-125b inhibitor and mimic were transfected into CNE2-IR and CNE2 cells, respectively, and then cell proliferation was determined by cell counting Kit-8 (CCK-8) assay, 5-ethynyl-2′-deoxyuridine (EdU) incorporation and plate colony formation assay. The results showed that miR-125b inhibitor significantly decreased, whereas miR-125b mimic significantly increased NPC cell proliferation as compared with control inhibitor or mimic (Figures 2b–d). Next, we analyzed the effect of miR-125b on the apoptosis of NPC cells by using flow cytometry. The results showed that miR-125b inhibitor significantly increased while miR-125b mimic significantly decreased the apoptosis of NPC cells as compared with control inhibitor or mimic (Figure 2e). Collectively, these results demonstrate that high miR-125b expression promotes NPC cell proliferation and inhibits NPC cell apoptosis *in vitro*.

### MiR-125b antagomir inhibits *in vivo* NPC cell growth

To determine the effect of miR-125b on *in vivo* NPC cell growth, we generated subcutaneous tumors in nude mice using CNE2-IR cells. Control or miR-125b antagomir was injected into the subcutaneous tumors, and then tumor growth was assessed. As shown in Figure 2f, growth of miR-125b antagomir-injected tumors was significantly lower than that of control antagomir-injected tumors as demonstrated by tumor growth and weight, demonstrating that inhibition of miR-125b expression reduces NPC xenograft tumor growth.

### MiR-125b promotes NPC cell proliferation and inhibits NPC cell apoptosis by targeting A20

**Figure 3:**
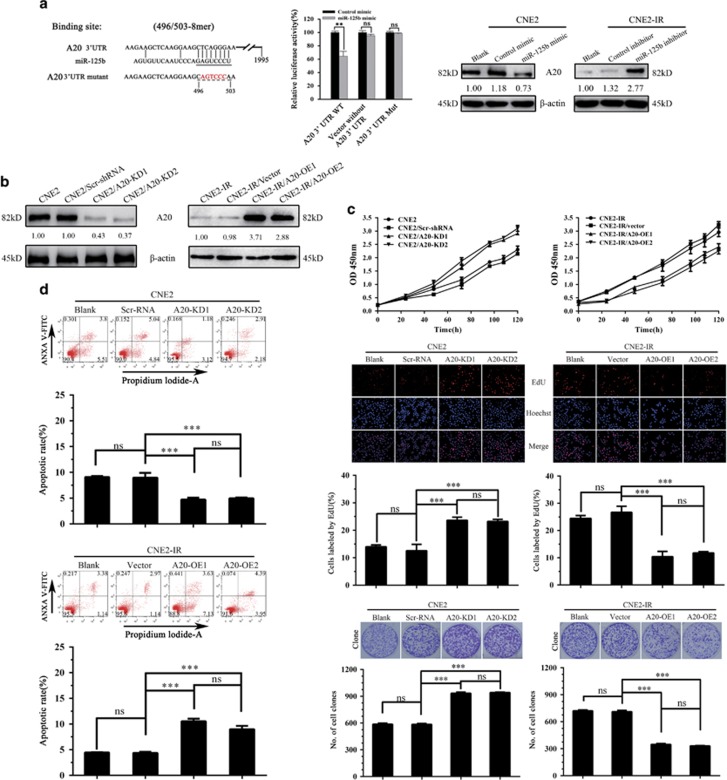
Target A20 of miR-125b regulates NPC cell proliferation and apoptosis. (**a**) 3′-UTR dual luciferase reporter assay showing A20 as a direct target of miR-125b in NPC cells. (left) The predicted miR-125b binding sites in the 3′-UTR of wild-type (wt) A20 and mutant (mt) A20 3′-UTR; (middle) Luciferase activity of wt and mt A20 3′-UTR and without A20 3′-UTR dual luciferase reporter vector in the CNE2 cells transfected with control or miR-125b mimic; (right) Western blot analysis showing A20 levels in the miR-125b mimic-transfected CNE2, miR-125 inhibitor-transfected CNE2-IR cells and their respective control cells. (**b**) Western blot analysis showing A20 levels in the A20 KD CNE2 cells, A20 OE CNE2-IR cells and their respective control cells. (**c**) Analysis of cell proliferation by CCK-8 (top), EdU incorporation (middle) and plate clone formation (bottom) assay in A20 KD CNE2 cells, A20 OE CNE2-IR cells and their respective control cells. (**d**) Analysis of cell apoptosis by flow cytometry in the A20 KD CNE2 cells, A20 OE CNE2-IR cells and their respective control cells. Three experiments were done; means, S.D.s, and statistical significance are denoted; **P<0.01; ***P<0.001; ns, no significance. Vector, transfected with an empty vector; KD, knockdown; OE, overexpression

To confirm A20 as a direct target of miR-125b, we co-transfected a dual luciferase reporter plasmid with wild-type A20 3′-UTR into CNE2 cells with control or miR-125b mimic. The results revealed a significant reduction in luciferase activity in miR-125b mimic-transfected cells compared with control mimic-transfected cells, whereas miR-125b mimic had no obvious effects on the luciferase activity of a dual luciferase reporter plasmid without A20 3′-UTR or with mutated A20 3′-UTR in the miR-125b-binding site (Figure 3a). Moreover, A20 level was significantly decreased in the miR-125b mimic-transfected CNE2 cells, whereas significantly increased in the miR-125b inhibitor-transfected CNE2-IR cells as compared with their respective control cells (Figure 3a). These results confirm that A20 is a direct target of miR-125b in NPC cells.

**Figure 4:**
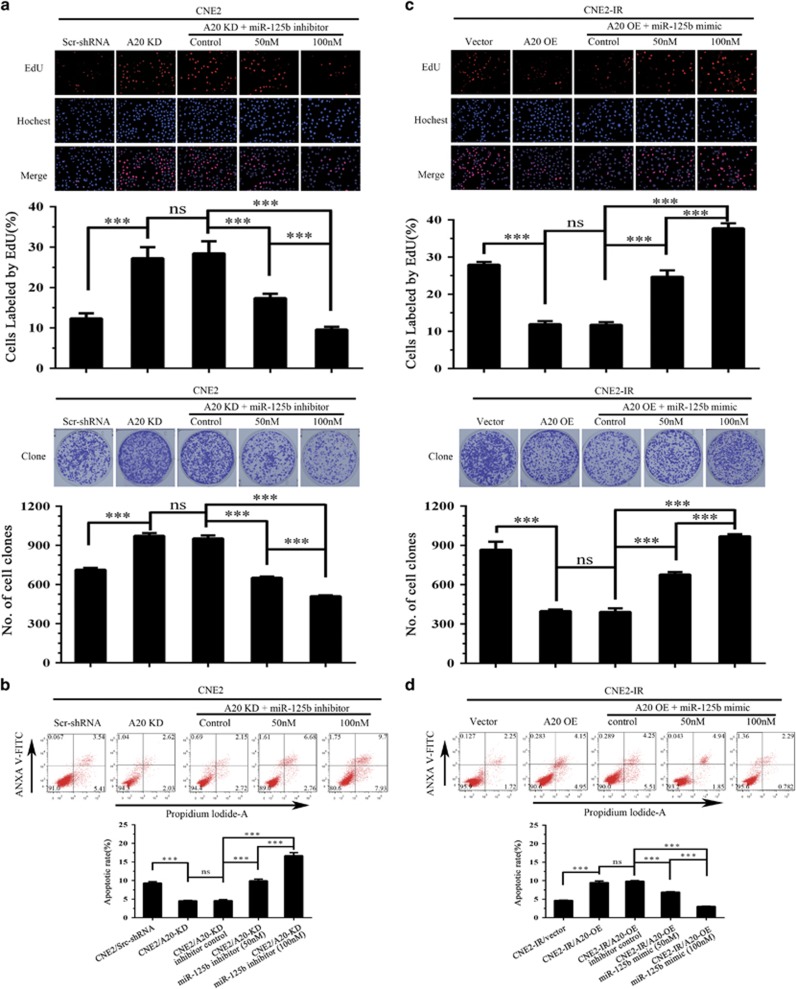
MiR-125b promotes NPC cell proliferation and inhibits NPC cell apoptosis by targeting A20. (**a**) Analysis of cell proliferation by EdU incorporation (top) and plate clone formation (bottom) assay in the A20 KD CNE2 cells transfected with miR-125b inhibitor and control cells. (**b**) Analysis of cell apoptosis by flow cytometry in the A20 KD CNE2 cells transfected with miR-125b inhibitor and control cells. (**c**) Analysis of cell proliferation by EdU incorporation (top) and plate clone formation (bottom) assay in the A20 OE CNE2-IR cells transfected with miR-125b mimic and control cells. (**d**) Analysis of cell apoptosis by flow cytometry in the A20 OE CNE2-IR cells transfected with miR-125b mimic and control cells. Three experiments were done; Means, S.D.s, and statistical significance are denoted; ***P<0.001; ns, no significance. Vector, transfected with an empty vector; KD, knockdown; OE, overexpression

Next, we analyzed whether A20 mediates miR-125b-regulating NPC cell proliferation and apoptosis. We established CNE2-IR cell lines with A20 overexpression (OE), CEN2 cell lines with A20 knockdown (KD) and their respective control cell lines (Figure 3b), and observed that A20 OE significantly decreased cell proliferation and induced cell apoptosis, whereas A20 KD significantly increased cell proliferation and inhibited cell apoptosis (Figures 3c and d), phenocopying those seen in the miR-125b inhibitor and mimic-transfected NPC cells, respectively. Moreover, A20 KD abolished the effects of miR-125b inhibitor on the proliferation and apoptosis of CNE2-IR cells (Figures 4a and b), and A20 OE abolished the effects of miR-125b mimic on the proliferation and apoptosis of CNE2 cells (Figures 4c and d). Taken together, our results demonstrate that miR-125 regulates NPC cell proliferation and apoptosis by targeting A20.

### A20 inhibits *in vivo* NPC cell growth

**Figure 5:**
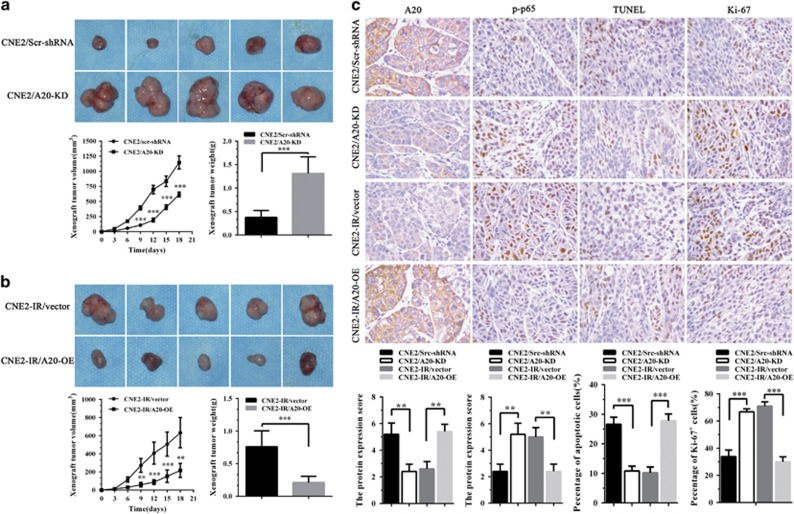
A20 inhibits *in vivo* NPC cell growth. (**a**) The representative photography of xenograft tumors after 18 days subcutaneous implantation of A20 KD CNE2 cells and control cells (top); Growth and weight of xenograft tumors generated by A20 KD CNE2 cells and control cells (bottom). (**b**) The representative photography of xenograft tumors after 18 days subcutaneous implantation of A20 OE CNE2-IR cells and control cells (top); Growth and weight of xenograft tumors generated by A20 OE CNE2-IR cells and control cells (bottom). (**c**) Representative results of A20, p-p65 (RelA), TUNEL, and Ki-67 immunohistochemical staining (top) and statistical analysis (bottom) of xenograft tumors generated by A20 KD CNE2 cells, A20 OE CNE2-IR cells and their respective control cells. *n*=10 mice per group. Original magnification, × 400. Means, S.D.s (*n*=10), and statistical significance are denoted; **P<0.01; ***P<0.001. Vector, transfected with an empty vector; KD, knockdown; OE, overexpression

Tumor formation assay in nude mice was performed to determine the effects of A20 on NPC cells growth *in vivo*. The results showed that A20 KD significantly increased, whereas A20 OE significantly decreased the growth of NPC xenograft tumors as demonstrated by tumor growth and weight (Figures 5a and b). Transferase-mediated dUTP nick end labeling (TUNEL) assay showed that A20 KD significantly decreased, whereas A20 OE significantly increased the number of apoptotic cells in the xenograft tumors (Figure 5c). Immunohistochemical staining showed that A20 KD significantly increased, whereas A20 OE significantly decreased the number of Ki-67 positive cells, that is, proliferation cells in the xenograft tumors (Figure 5c). Moreover, A20 KD significantly increased, whereas A20 OE significantly decreased the expression of p-p65 (RelA) in the xenograft tumors (Figure 5c). Collectively, these results suggest that A20 inhibits *in vivo* NPC cells growth possibly through inhibiting cells proliferation and inducing cell apoptosis, supporting that miR-125b regulates NPC cell proliferation and apoptosis by targeting A20.

### NF-κB mediates miR-125b/A20-regulating NPC cell proliferation and apoptosis

**Figure 6:**
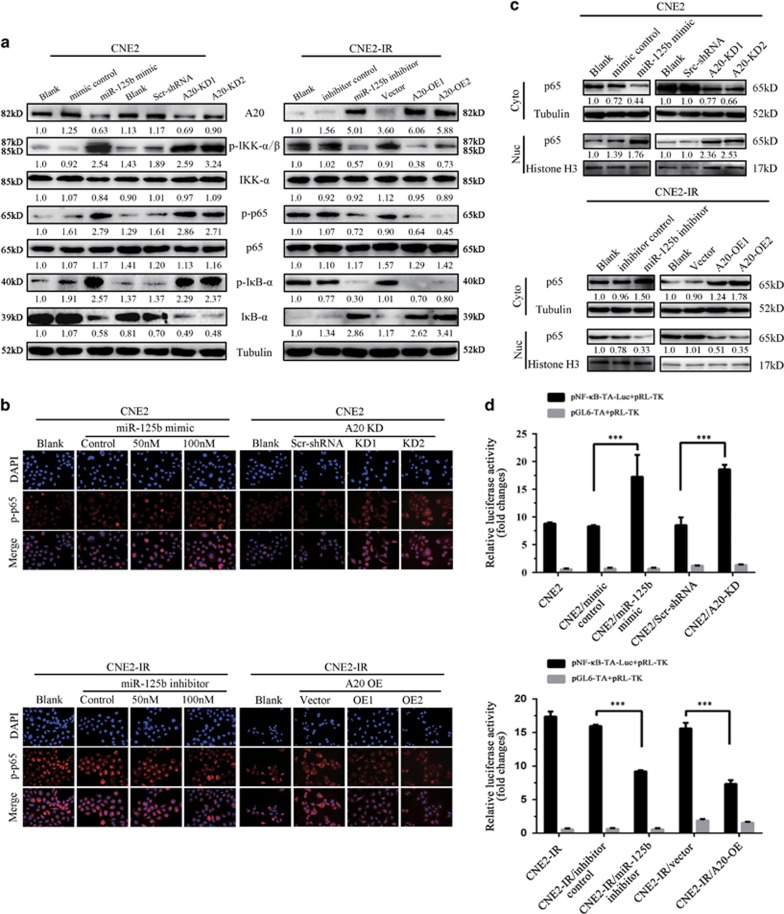
MiR-125b regulates the activity of NF-*κ*B signaling pathway by targeting A20 in NPC cells. (**a**) Western blot analysis showing the levels of p-IKK*α*/*β*, p-I*κ*B*α*, p-p65(RelA), IKK*α*/*β*, I*κ*B*α*, and p65 in the miR-125b mimic-transfected CNE2 cells, miR-125 inhibitor-transfected CNE2-IR cells, A20 KD CNE2 cells, A20 OE CNE2-IR cells, and their respective control cells. (**b**) Representative immunofluorescent staining showing the nuclear translocation of p-p65(RelA) in the miR-125b mimic-transfected CNE2 cells, miR-125 inhibitor-transfected CNE2-IR cells, A20 KD CNE2 cells, A20 OE CNE2-IR cells, and their respective control cells. (**c**) Western blot analysis showing p65(RelA) expression in the nuclear and cytoplasmic fractions of miR-125b mimic-transfected CNE2 cells, miR-125 inhibitor-transfected CNE2-IR cells, A20 KD CNE2 cells, A20 OE CNE2-IR cells, and their respective control cells. Nuc, nuclear fraction; Cyt, cytoplasmic fraction. (**d**) A luciferase reporter assay showing p65 transcriptional activity in the miR-125b mimic-transfected CNE2 cells, miR-125 inhibitor-transfected CNE2-IR cells, A20 KD CNE2 cells, A20 OE CNE2-IR cells, and their respective control cells. Three experiments were done; Means, S.D.s and statistical significance are denoted; ***P<0.001; ns, no significance. Vector, transfected with an empty vector; KD, knockdown; OE, overexpression

**Figure 7:**
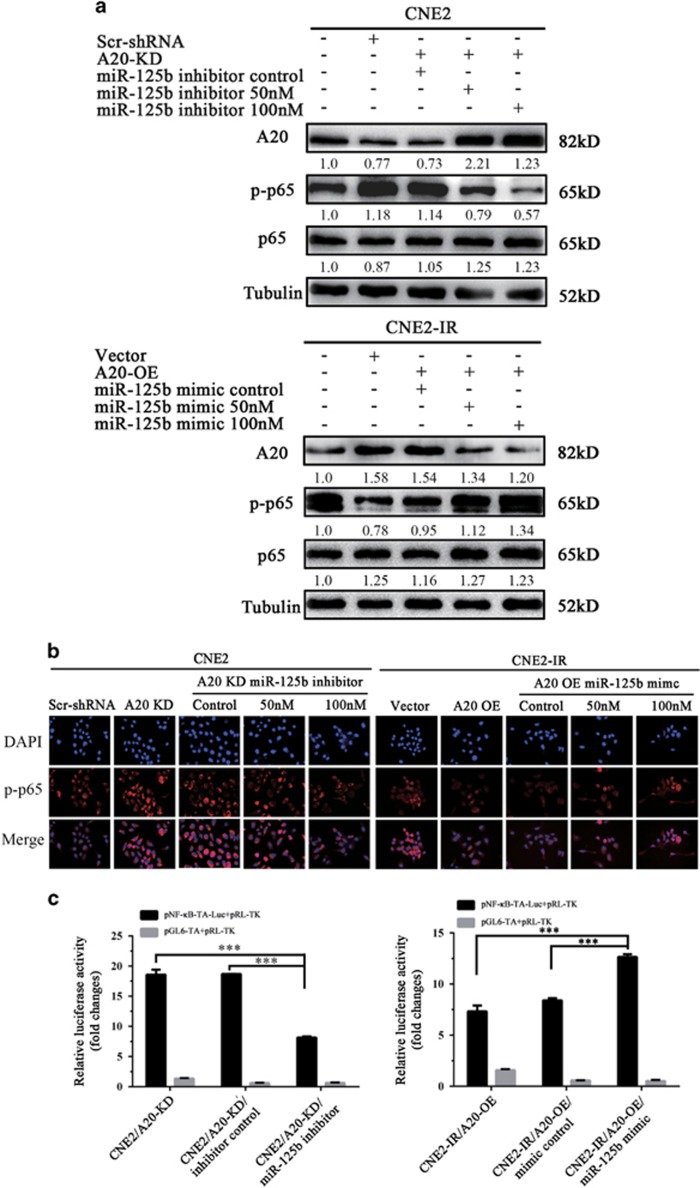
The effect of A20 expression changes on miR-125b-regulating NF-*κ*B activity. Western blot analysis showing p-p65(RelA) levels (**a**), representative immunofluorescent staining showing the nuclear translocation of p-p65 (**b**), and luciferase reporter assay showing p65 transcriptional activity (**c**) in the A20 KD CNE2 cells transfected with miR-125b inhibitor, A20 OE CNE2-IR cells transfected with miR-125b mimic, and their respective control cells. Three experiments were done; Means, S.D.s and statistical significance are denoted; ***P<0.001. Vector, transfected with an empty vector; KD, knockdown; OE, overexpression

Activation of NF-*κ*B signaling pathway has a crucial role in the development and progression of NPC,^9, 10, 11, 12^ and A20 is a negative regulator of NF-*κ*B signaling pathway.^15, 16, 17, 18, 19^ Therefore, we investigated whether NF-*κ*B mediates miR-125b/A20-regulating NPC cell proliferation and apoptosis. The results showed that either miR-125b mimic or A20 KD significantly enhanced the phosphorylated levels of IKK*α*/*β*, I*κ*B*α*, and p65, the nuclear translocation of p-p65 and NF-*κ*B luciferase reporter activity in the CNE2 cells, whereas either miR-125b inhibitor or A20 OE significantly reduced the phosphorylated levels of IKK*α*/*β*, I*κ*B*α*, and p65, the nuclear translocation of p-p65 and NF-*κ*B luciferase reporter activity in the CNE2-IR cells (Figures 6a–d). Importantly, A20 OE abrogated activity of NF-*κ*B increased by miR-125b inhibitor in the CNE2 cells, and A20 KD restored activity of NF-*κ*B decreased by miR-125b mimic in the CNE2-IR cells (Figures 7a–c). Collectively, these results demonstrate that miR-125b regulates the activity of NF-*κ*B signaling pathway by targeting A20.

**Figure 8:**
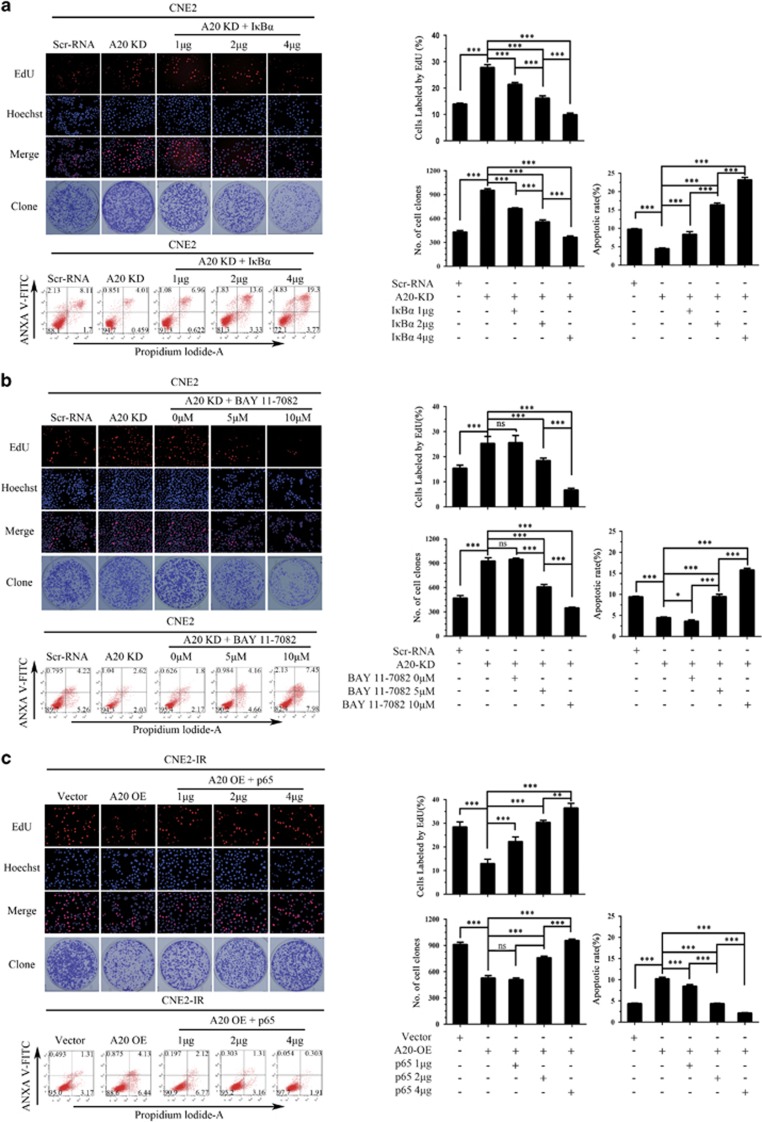
NF-κB mediates miR-125b/A20-regulating NPC cell proliferation and apoptosis. (**a**) Representative results (left) and statistical analyses (right) of EdU incorporation assay, plate clone formation assay and detection of cell apoptosis by flow cytometry in the A20 KD CNE2 cells transfected with plasmid expressing IκB*α* and control cells. (**b**) Representative results (left) and statistical analyses (right) of EdU incorporation assay, plate clone formation assay and detection of cell apoptosis by flow cytometry in the A20 KD CNE2 cells treated with BAY11-7082 and control cells. (**c**) Representative results (left) and statistical analyses (right) of EdU incorporation assay, plate clone formation assay and detection of cell apoptosis by flow cytometry in the A20 OE CNE2-IR cells transfected with plasmid expressing p65 and control cells. Three experiments were done; Means, S.D.s, and statistical significance are denoted; *P<0.05; ***P<0.001; ns, no significance. Vector, transfected with an empty vector; KD, knockdown; OE, overexpression

Next, we determined whether NF-κB mediates miR-125b/A20-regulating NPC cell proliferation and apoptosis. We observed that either exogenous I*κ*B*α* OE or NF-*κ*B inhibitor BAY11-7082 treatment significantly abolished the effects of A20 KD on NPC cell proliferation and apoptosis, whereas exogenous p65 OE restored the effects of A20 OE on NPC cell proliferation and apoptosis (Figures 8a–c). These results demonstrate that NF-κB signaling mediates miR-125b/A20-regulating NPC cell proliferation and apoptosis.

### Levels of miR-125b, A20, and p-p65 are correlated in human NPC biopsies

As our data demonstrate that miR-125b regulates NPC cell proliferation and apoptosis by targeting A20/NF-κB, we next determined whether the levels of miR-125b, A20 and p-p65 were correlated in human NPC biopsies. Our immunohistochemistry showed that A20 expression was significantly lower, whereas p-p65 was significantly higher in the NPC tissues than that in the NNM (Figure 1c, Table 1). Correlation analyses revealed that miR-125b level was negatively associated with A20 level (*r*=−0.61, *P*<0.001), whereas positively associated with p-p65 level (*r*=0.45, *P*<0.001), and A20 level was negatively associated with p-p65 level (*r*=−0.38, *P*<0.001). Together, these results indicate that high miR-125b expression appears to be associated with downregulation of A20 and the activations of NF-*κ*B in the NPC tissues, and these misregulations might contribute to NPC development.

## Discussion

The dysregulation of miRNA has been implicated in human cancers.^2, 3^ In this study, we focused on miR-125b, because the role and mechanism of miR-125b in NPC are unclear. We observed that miR-125b was frequently upregulated in the NPC tissues relative to NNM, and its increment correlated with poor patient survival, outlining a potential marker for predicting the prognosis of NPC patients. To gain insight into miR-125b function in the pathogenesis of NPC, we analyzed the effects of miR-125b on NPC cell proliferation and apoptosis, and found that downregualtion of miR-125b decreased cell proliferation and induced cell apoptosis, whereas upregualtion of miR-125b increased cell proliferation and inhibited cell apoptosis, suggesting that miR-125b acts as oncogene in NPC.

As miRNAs exert their roles through inhibiting target mRNA translation, thus identification of miR-125b target genes is a key step for understanding the mechanism of miR-125b-regulating NPC cell proliferation and apoptosis. It has been reported that miR-125b activates NF-*κ*B pathway by targeting A20,^20, 21, 22^ and activation of NF-*κ*B signaling confers the advantages of tumor cell proliferation and survival.^8^ Therefore, we analyzed whether miR-125b regulates NPC cell proliferation and apoptosis by targeting A20. Our results confirm that A20 is a direct target of miR-125b in NPC cells, and miR-125b regulates NPC cell proliferation and apoptosis by targeting A20.

Numerous studies have revealed that A20 negatively regulates NF-*κ*B signaling pathway by inhibiting the function of several NF-*κ*B upstream signaling transducers.^15, 16, 17, 18, 19 and 20^ Activation of NF-*κ*B signaling pathway has a crucial role in the development and progression of NPC.^9, 10, 11 and 12^ Therefore, we investigated whether A20 mediates miR-125b-regulating NPC cell proliferation and apoptosis by activating NF-*κ*B. We observed that miR-125b mimic or A20 KD enhanced, whereas miR-125b inhibitor or A20 overexpression reduced the activity of NF-*κ*B signaling pathway in NPC cells. Importantly, A20 overexpression abrogated activation of NF-*κ*B signaling induced by miR-125b mimic, and A20 KD restored activity of NF-*κ*B signaling decreased by miR-125b inhibitor in the NPC cells. These results demonstrate that miR-125b activates NF-*κ*B by targeting A20. We further showed that either I*κ*Ba overexpression or BAY11-7082 abolished the effects of A20 KD on NPC proliferation and apoptosis, whereas p65 (RelA) overexpression abolished the effects of A20 overexpression on NPC cell proliferation and apoptosis. In the clinical NPC samples, p-p65 (RelA) level was negatively associated with A20 level, whereas positively associated with miR-125b level. Taken together, our results demonstrate that NF-kB signaling mediates miR-125b/A20-regulating NPC cell proliferation and apoptosis.

Numerous studies have indicated that A20 is involved in the pathogenesis of various types of lymphoid malignancies,^24, 25, 26, 27, 28^ multiple myeloma^29, 30^ and non-small cell lung cancer^31^ duo to its genetic or epigenetic inactivation, leading to A20 downregulation, which functions as a tumor suppressor. It is also reported that A20 acts as oncogene in glioblastoma,^32, 33^ hepatitis B virus-related hepatocellular carcinoma,^34^ and gastric cancer.^35^ However, the expression and function of A20 in NPC are poorly understood. In this study, we observed that A20 was significantly decreased in NPC tissues relative to NNM, inhibited NPC cell proliferation, and induced NPC apoptosis; A20 KD significantly increased while A20 ovexpression significantly decreased the growth of NPC xenograft tumors in nude mice possibly through inhibiting cell proliferation and inducing cell apoptosis. The results strongly suggest that A20 functions as a tumor suppressor in NPC.

Although A20/NF-*κ*B signaling axis seems to largely account for the phenotype of NPC cells induced by miR-125b, indeed a single miRNA can target multiple mRNAs to regulate gene expression.^36^ Therefore, there might be other molecules such as BAK1, PPP1CA, and p53,^37, 38, 39, 40, 41^ which are also targeted by miR-125b in NPC cells. We also observed that miR-125b negatively modulated p53 expression in NPC cells, but it still promoted cell proliferation and inhibited cell apoptosis in p53 KD NPC CNE2 cell line that was established previously by us (Supplementary Figure 1).^42^ The results suggest that miR-125b promotes NPC cell proliferation and inhibits NPC cell apoptosis by p53-independent manner.^43^

In summary, our data demonstrate that miR-125b is frequently upregulated in the NPC biopsies, and is an independent predictor for NPC patient survival; miR-125b regulates NPC cell proliferation and apoptosis by targeting A20/NF-*κ*B signaling pathway; A20 inhibits NPC cell proliferation and induces NPC cell apoptosis both *in vitro* and growth i*n vivo*. Our data demonstrate that miR-125b act as an oncogene, whereas A20 act as a tumor suppressor in NPC, and suggests that targeting miR-125b/A20/NF-*κ*B signaling axis is a promising approach for NPC patients with high miR-125b expression.

## Materials and Methods

### Patients and tissue samples

A total of 111 NPC patients without distant metastasis (M0 stage) at the time of diagnosis who were treated by radical radiotherapy alone in the Affiliated Cancer Hospital of Central South University, China between January 2006 and December 2008 were recruited in this study. The radiotherapy was administered for a total dose of 60–70 Gy (2 Gy/fraction, 5 days a week). The neck received 60Gy for lymph node-negative cases and 70 Gy for lymph node-positive cases. NPC tissue biopsies were obtained at the time of diagnosis before any therapy, fixed in 4% formalin and embedded in paraffin. We also acquired 30 cases of formalin-fixed and paraffin-embedded NNM in the same period. On the basis of the 1978 WHO classification,^44^ all tumors were histopathologically diagnosed as poorly differentiated squamous cell carcinomas (WHO type III). The clinical stage of the patients was classified according to the 2008 NPC staging system of China.^45^

The patients were followed up, and the follow-up period at the time of analysis was >72 months (average, 77.5±11.8 months). DFS was calculated as the time from the completion of primary radiotherapy to the date of pathological diagnosis or clinical evidence of local failure and/or distant metastasis. OS was defined as the time from the initiation of primary radiotherapy to the date of cancer-related death or when censured at the latest date if patients were still alive. The clinicopathologic parameters of the patients used in the present study are shown in Supplementary Table S2.

### Cell lines

Human NPC cell lines CNE2-IR and CNE2 cells have been described previously by us,^23^ and cultured with RPMI-1640 medium supplemented with 10% fetal bovine serum (Life Technologies, Carlsbad, CA, USA) at 37 °C in 5% CO_2_. The cell lines were routinely tested for presence of mycoplasma with 4,6-diamidino-2-phenylindole staining, and were mycoplasma free.

### QRT-PCR

QRT-PCR was performed as described previously by us.^23^ In brief, total RNA was extracted from NPC cells with Trizol reagent (Invitrogen, Carlsbad, CA, USA), or from paraffin-embedded NPC and NNM with RecoverAll total nucleic acid isolation kit (Ambion, Austin, TX, USA) according to the manufacturer’s instructions. For miR-125b qRT-PCR, 2 *μ*g of total RNA was reversely transcribed for cDNA using a reverse transcription (RT) kit according to the manufacturer’s instructions (Promega, Madison, WI, USA) and miR-125b specific primer (Bulge-Loop miRNA qPCR primer). The RT products were amplified by real-time PCR using miScript SYBR green PCR kit (Qiagen, Hilden, Germany) according to the manufacturer’s instructions. For A20 mRNA qRT-PCR, 2 *μ*g of total RNA was reversely transcribed for cDNA using a RT kit according to the manufacturer’s protocol and Oligo dT primer (Promega) according to the manufacturer’s instructions. The RT products were amplified by real-time PCR using QuantiFast SYBR green PCR kit (Qiagen) according to the manufacturer’s instructions. The products were quantitated using 2^-*DDCt*^ method against U6 or GAPDH for normalization. The primer sequences were synthesized by RiboBio (Guangzhou, China) and summarized in Supplementary Table S3.

### Luciferase activity assay

For the A20 3′-UTR luciferase reporter assay, a dual luciferase reporter plasmid with wild-type A20 3′-UTR (HmiT018145-MT01, GeneCopoeia), without A20 3′-UTR (CmiT000001-MT01, GeneCopoeia), or with mutated A20 3′-UTR in the predicted miR-125b binding site constructed by GeneCopoeia, and miR-125b or control mimic (RiboBio) were co-transfected into NPC cells using Lipofectamine 2000 (Invitrogen) according to the manufacturer’s instructions. For the NF-κB luciferase reporter assay, a reporter plasmid containing human NF-κB/p65 response element (pNF-κB-TA-luc) (Beyotime, Nanjin, China) or pGL6-TA plasmid without NF-κB response element (Beyotime) and pRL-TK plasmid (Promega) were co-transfected into the indicated NPC cells using Lipofectamine 2000. Cells were harvested 48 h after transfection, both firefly luciferase and renilla luciferase activities were measured using the dual luciferase reporter assay system (Promega) according to the manufacturer’s instructions, and luciferase activity was estimated using a luminometer (Promega).

### Transfection of miR-125b mimic and inhibitor into cells

A total of 50 and 100 nmol/ml miR-125b mimic, miR-125b inhibitor and their respective negative control (Ribobio) were transfected into the indicated NPC cells using RiboFect CP transfection kit (Ribobio) according to the manufacturer’s instructions, respectively. Twenty-four hours after transfection, cells were subjected to further analysis.

### Establishment of NPC cell lines with A20 OE and KD

Lentiviral GV248-A20 shRNA and GV248-scramble non-target shRNA vector, which was established by Genechem (Shanghai, China), and confirmed by sequencing. The target for human A20 shRNA was 5′-CACTGGAAGAAATACACATAT-3′, the KD efficiency of which has been validated.^33^ A20 expression plasmid (EX-K6040) and control vector pReceiver-M13 were purchased from GeneCopoeia. Cells were infected or transfected with the lentiviral vectors or plasmids according to the manufacturer’s instructions, and then selected using neomycin or puromycin for 2 weeks. NPC cell lines with stable KD or OE of A20 and control cell lines were obtained.

### CCK-8 assay

Cell proliferation was measured using a CCK-8 kit. In brief, the cells were plated at 1 × 10^3^ cells/well in 96-well tissue culture plates, and grew for 7 days. Every 24 h, 10 *μ*l CCK-8 reagent (Beyotime) was added to every well, and incubated for 4 h. The absorbance of each well was read with a Bio-Tek Instruments EL310 Microplate Autoreader at 450 nm. CCK-8 assay was performed three times in triplicate.

### EdU incorporation assay

Cell proliferation was measured using EdU assay. In brief, the cells were cultured in chamber slides (Millipore, Billerica, MA, USA; 2 × 10^4^ cells/well). Forty-eight hours after culture, the cells were treated with 50 *μ*M EdU (RiboBio) for an additional 2 h at 37 °C, and then were fixed with 4% formaldehyde for 30 min, followed by addition of 200 *μ*l glycine (2 mg/ml; Amresco, OH, USA). After 5 min, the cells were incubated with 0.5% Triton X-100 (Sigma-Aldrich, St. Louis, MO, USA) for 10 min at room temperature. Following washing with PBS for 5 min, 1 × Apollo reaction reagent (RiboBio) was added and incubated at room temperature in the dark for 30 min, and then the cells were stained with 200 *μ*l Hoechst 33342 (5 *μ*g/ml; Sigma-Aldrich) for an additional 30 min in the dark. Cells labeled and unlabeled by EdU were counted under a Leica DMI4000 microscope, and pictures were taken. The assay was performed three times in triplicate.

### Plate colony formation assay

Plate colony formation assay was performed to detect cell proliferation. In brief, 1 × 10^3^cells were plated into each well of six-well plate, and were growing for 14 days. At the end of the experiment, cell colonies were fixed with 4% paraformaldehyde, and stained with 0.5% crystal violet (Sigma-Aldrich) stained. The numbers of colony were counted under a Leica DMI4000 microscope, and pictures were taken. The assay was performed three times in triplicate.

### Analysis of cell apoptosis by flow cytometry

Cell apoptosis was assessed by Annexin V-fluorescein isothiocyanate apoptosis detection kit I (BD Biosciences, San Diego, CA, USA) according to the manufacturer’s instructions. In brief, 5 × 10^5^ cells were collected by centrifugation, resuspended in 500 *μ*l binding buffer, and stained with 5 *μ*l Annexin V conjugated with fluorescein isothiocyanate and 5 *μ*l propidium iodide at room temperature in the dark for 15 min, and then immediately analyzed by a FACSCalibur System. The relative proportion of Annexin V-positive cells was determined using the CellQuest Pro software and counted as the percentage of apoptotic cells. The assay was performed in triplicate for three times.

### Tumor formation assay in nude mice

Nude male mice that were 4 weeks old were obtained from the Laboratory Animal Center of Central South University (Changsha, China) and were maintained under specific pathogen-free conditions. For tumor formation experiment, 5 × 10^6^ cells resuspended in 200 *μ*l of medium without serum were subcutaneously injected into the flanks of mice (*n*=10 mice each). The mice were monitored daily for palpable tumor formation, and tumor volume (in mm^3^) was measured by a Vernier caliper every 3 days and calculated by using the modified ellipse formula (volume=length × width^2^/2). At the end of the experiments, the mice were killed by cervical dislocation, and tumors were excised, weighted, and embedded in paraffin for immunohistochemical staining.

To test the effect of miR-125b on *in vivo* NPC cells growth, 5 × 10^6^ CNE2-IR cells were subcutaneously injected into the flanks of mice. When the xenograft volumes reached approximately 50 mm^3^, the transplanted mice were randomly divided into two groups (*n*=5 mice each), 20 nmol control or miR-125 antagomir (RiboBio) in 25 μl saline buffer was intratumorally injected into the tumor mass at multiple sites per mouse, and then tumor growth was monitored as above.

### Western blot

Total proteins were exacted from cells as described previously by us.^23^ Nuclear and cytoplasmic proteins were prepared by using nuclear and cytoplasmic protein extraction kit (#P0027, Beyotime) according to the manufacturer’s instructions. An equal amount of protein in each sample was subjected to SDS-PAGE separation, followed by blotting onto a PVDF membrane. After blocking, blots were incubated with antibodies against A20 (ab92324, Abcam, Cambridge, MA, USA), p-IKK*α*/*β* (#2078, CST), p-IκB*α* (#2859, CST), p-p65(RelA) (#3033, CST), IKK*α* (#2682, CST), I*κ*B*α* (#4812, CST), p65(RelA) (#4764, CST), or p53 (sc-47698, Santa Cruz, CA, USA) overnight at 4°C, followed by incubation with HRP-conjugated secondary antibody (#A24531 or #A24512, Life Technologies) for 2 h at room temperature. The signal was visualized with an enhanced chemiluminescence detection reagent (Pierce). Tublin (ab15246, Abcom), *β*-actin (sc-1616, Santa Cruz) or Histone H3 (ab8580, Abcom) was simultaneously detected as a loading control. The assays were performed for three times.

### Immunohistochemistry

Immunohistochemical staining of A20, p-p65(RelA), and Ki-67 were performed on the formalin-fixed and paraffin-embedded tissue sections as described previously by us.^46^ In brief, after antigen retrieval tissue sections were incubated with antibodies against A20, p-p65(RelA), or -Ki-67 antibody (ab15580, Abcom) overnight at 4 °C, and then were incubated with biotinylated secondary antibody followed by avidin-biotin peroxidase complex. Finally, tissue sections were incubated with 3′, 3′-diaminobenzidine (Sigma-Aldrich) and counterstained with hematoxylin. In negative controls, primary antibodies were replaced with a normal mouse or rabbit IgG. Known immunostaining-positive slides were used as positive controls.

The immunoreactions were evaluated independently by two pathologists as described previously by us.^46^ A staining score of ⩽3 was considered to be low expression; and a score of >3 was considered to be high expression. Quantitative evaluation of proliferation cells was done by examining the sections in five random microscopic fields and counting the number of Ki-67 positive nuclei among 1000 cancer cells under the light microscope. The rate of proliferation cells was expressed as positive cells per 100 cancer cells.

### *In situ* detection of apoptotic cells

Terminal deoxynucleotidyl TUNEL was performed to detect apoptotic cells of formalin-fixed and paraffin-embedded tissue sections of xenograft tumors with *in situ* cell death detection kit (Roche Applied Science, Shanghai, China) according to the manufacturer’s instructions. Quantitative evaluation of apoptotic cells was done by examining the sections in five random microscopic fields and counting the number of TUNEL-positive cancer cells among 1000 carcinoma cells under the light microscope. The apoptotic index was expressed as positive cells per 100 cancer cells.

### Immunofluorescent staining

A total of 2 × 10^3^ cells were plated into chamber slides and cultured with RPMI-1640 medium containing 10% FBS for 12 h. Cells were fixed with 4% paraformaldehyde at room temperature for 15 min, and then cell membranes were permeabilized with 0.1% Triton 100 at room temperature for 20 min. Cells were washed with 1 × PBS and blocked with 10% goat serum in PBS for 1 h. Then cells were incubated with primary antibody against p-p65 (RelA) overnight at 4 °C. After washing with 1 × PBS for three times, cells were incubated with secondary antibodies conjugated with Alexa Fluor 594 (DI-1794, Vector, Burlingame, CA, USA) for 1 h. The slides were washed three times with 1 × PBS, counterstained with DAPI (Vector Laboratories), mounted and stored at 4 °C under dark conditions. Pictures were taken under a Leica DMI4000 microscope.

### Statistical analysis

All experiments were carried out at least three times. Data were presented as the mean±S.D. Statistical analysis was conducted using SPSS 22.0 statistical software package. For comparisons between two groups, a Student *t*-test or *χ*^2^***-***test was used, and for analysis with multiple comparisons, one-way ANOVA test or Wilcoxon and Mann–Whitney test was used. Survival curves were obtained by using Kaplan–Meier method, and comparisons were made by using log-rank test. Univariate and multivariate survival analyses were conducted on all parameters by using Cox proportional hazards regression model. The Spearman rank correlation coefficient was used to determine the correlation between two parameters. All statistical tests were two-sided. *P*-values <0.05 were considered to be statistically significant.

### Ethics statement

This study was approved by the ethics committee of Xiangya School of Medicine, Central South University, China. Written informed consent was obtained from all participants in the study. All animal experiments were undertaken in accordance with the Guide for the Care and Use of Laboratory Animals of Central South University, with the approval of the Scientific Investigation Board of Central South University.

## Figures and Tables

**Table 1:** The expression levels of A20 and phospho-p65 in the NNM and NPC tissues

	**NNM (*n*, %)**	**NPC (*n*, %)**	***P** (****χ****^**2**^***-test)**
*A20*			
Low (0–3)	7 (23.33)	68 (61.26)	<0.001
High (4–6)	23 (76.67)	43 (38.74)	

*p-p65*			
Low (0–3)	28 (93.33)	36 (32.43)	<0.001
High (4–6)	2 (6.67)	75 (67.57)	

Abbreviations: NNM, normal nasopharyngeal mucosa; NPC, nasopharyngeal carcinoma.
