# Supplementary material for: MiR-125b regulates proliferation and apoptosis of nasopharyngeal carcinoma by targeting A20/NF-κB signaling pathway
Source: Cell Death Dis. 2017 Jun 1;8(6):e2855–. doi: 10.1038/cddis.2017.211 (PMC5520883; doi:10.1038/cddis.2017.211)
Supplement: Supplementary Tables [file cddis2017211x1.doc]

**Supplementary Table S1. Univariate and multivariate analyses of prognostic factors for overall and disease-free survival using Cox proportional hazards regression model (N =111)**

| **Variable** | **Disease-free survival** | | | | |  | **Overall survival** | | | | |
| --- | --- | --- | --- | --- | --- | --- | --- | --- | --- | --- | --- |
|  | **Univariate analysis** | |  | **Multivariate analysis** | |  | **Univariate analysis** | |  | **Multivariate analysis** | |
|  | **HR** | **95% CI** |  | **HR** | **95% CI** |  | **HR** | **95% CI** |  | **HR** | **95% CI** |
| **Age( y)** |  |  |  |  |  |  |  |  |  |  |  |
| ≥ 46 | 1.000 |  |  | 1.000 |  |  | 1.000 |  |  | 1.000 |  |
| < 46 | 0.824 | 0.490~1.387 |  | 0.876 | 0.516~1.488 |  | 0.817 | 0.463~1.442 |  | 0.834 | 0.471~1.476 |
| **Gender** |  |  |  |  |  |  |  |  |  |  |  |
| Male | 1.000 |  |  | 1.000 |  |  | 1.000 |  |  | 1.000 |  |
| Female | 0.765 | 0.385~1.530 |  | 0.768 | 0.384~1.535 |  | 0.925 | 0.445~1.922 |  | 0.932 | 0.448~1.938 |
| **Primary tumor (T)stage** |  |  |  |  |  |  |  |  |  |  |  |
| T1~2 | 1.000 |  |  | 1.000 |  |  | 1.000 |  |  | 1.000 |  |
| T3~4 | 0.801 | 0.465~1.378 |  | 0.881 | 0.502~1.547 |  | 0.980 | 0.542~1.770 |  | 1.024 | 0.559~1.876 |
| **Lymph node (N) metastasis** |  |  |  |  |  |  |  |  |  |  |  |
| N0 | 1.000 |  |  | 1.000 |  |  | 1.000 |  |  | 1.000 |  |
| N1~3 | 2.037 | 0.882~4.706 |  | 2.085 | 0.898~4.841 |  | 1.954 | 0.841~4.536 |  | 1.931 | 0.830~4.495 |
| **Clinical TNM staging** |  |  |  |  |  |  |  |  |  |  |  |
| I ~ II | 1.000 |  |  | 1.000 |  |  | 1.000 |  |  | 1.000 |  |
| III ~ IVa | 5.252^#^ | 1.790~15.407 |  | 5.616^$^ | 2.026~15.568 |  | 4.952^*^ | 1.446~16.954 |  | 5.770^#^ | 1.787~18.623 |
| **MiR-125b expression level** |  |  |  |  |  |  |  |  |  |  |  |
| Low | 1.000 |  |  | 1.000 |  |  | 1.000 |  |  | 1.000 |  |
| High | 1.873^*^ | 1.146~3.060 |  | 1.816^*^ | 1.119~2.947 |  | 1.360^*^ | 1.086~2.353 |  | 1.372^*^ | 1.094~2.371 |

**P* < 0.05, ^#^*P* < 0.01, ^$^*P* < 0.001. HR, hazard ratio; CI, confidence interval.

**Supplementary Table S2. The clinicopathological parameters of 111 patients with nasopharygeal carcinoma**

| **Variable** | **No. of patients** | **%** |
| --- | --- | --- |
| **Gender**  Male | 88 | 79.28 |
| Female | 23 | 20.72 |
| **Age** |  |  |
| ≥46 | 52 | 46.85 |
| <46 | 59 | 53.15 |
| **Primary tumor (T) stage** |  |  |
| T1-2 | 62 | 55.86 |
| T3-4 | 49 | 44.14 |
| **Lymph node (N) metastasis** |  |  |
| N0 | 23 | 20.72 |
| N1-3 | 88 | 79.28 |
| **Clinical TNM stage** |  |  |
| I-II | 25 | 22.52 |
| III-IVa | 86 | 77.48 |

**Supplementary Table S3. QRT-PCR primers for amplification of miR-125b and A20 synthesized by Ribobio**

| **No.** | **Name** | **RT primer** | **Primer sequence** | |
| --- | --- | --- | --- | --- |
| 1 | miR-125b | Cat.#, SSD809230075 | F: Cat.#, SSD809230767 |  |
|  |  |  | R: Cat.#, SSD0892261711 |  |
| 2 | U6 | Cat.#, SSD0904071008 | F: Cat.#,SSD0904071006 |  |
|  |  |  | R: Cat.#,SSD0904071007 |  |
| 3 | A20 | Oligo dT | F: 5’- AGTTTCGGGAGATCATCCAC-3’ |  |
|  |  |  | R: 5’- TTGCCGTCACCGTTCGT-3’ |  |
| 4 | GAPDH | Oligo dT | F: 5’-TGACTTCAACAGCGACACCCA-3’ |  |
|  |  |  | R: 5’-CACCCTGTTGCTGTAGCCAAA-3’ |  |
