# Supplementary figures and images for: MiR-125b regulates proliferation and apoptosis of nasopharyngeal carcinoma by targeting A20/NF-κB signaling pathway
Source: Cell Death Dis. 2017 Jun 1;8(6):e2855–. doi: 10.1038/cddis.2017.211 (PMC5520883; doi:10.1038/cddis.2017.211)

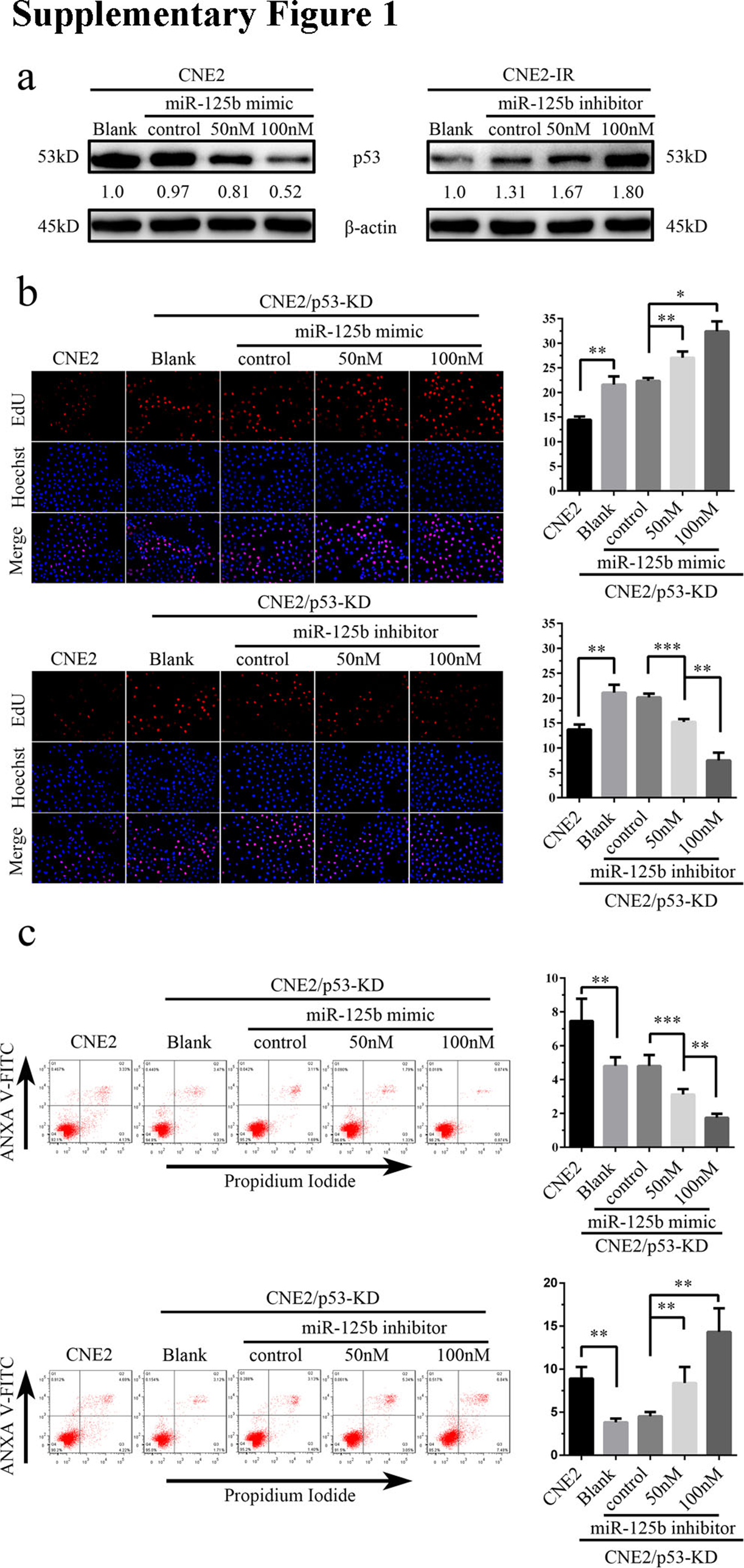

Supplement: Supplementary Figure 1 [file cddis2017211x2.tif]
